# The heat shock factor Cbr-HSF-1 is necessary for lifespan maintenance in *C. briggsae*

**DOI:** 10.17912/micropub.biology.002156

**Published:** 2026-06-27

**Authors:** Harvir Bhullar, Jordan Dias, Khushi Pastagia, Wouter van den Berg, Bhagwati P Gupta

**Affiliations:** 1 Biology, McMaster University, Hamilton, ON, CA

## Abstract

The heat shock response is a conserved mechanism that plays a critical role in organismal survival under thermal stress. *Caenorhabditis briggsae* is a widely used model organism for comparative studies involving its well-known cousin *C. elegans* and exhibits increased thermotolerance under heat stress. In *C. elegans* , the transcription factor HSF-1 has been well characterized for its role in many processes including development, thermotolerance, and lifespan. We recently showed that *Cbr- hsf-1* is necessary for fertility and protection against heat stress. Here, we report that *Cbr- hsf-1* also plays essential roles during development and adulthood to maintain the lifespan of animals.&nbsp;

**Figure 1. Survival and stress response of C. briggsae following hsf-1 RNAi knockdown:** **(A)** Schematic of RNAi treatment timelines used for lifespan assays. **(B-D)** Lifespan curves of animals. Control animals were fed bacteria carrying an empty vector (L4440). RNAi was administered from **(B)** embryogenesis to Day 1 adulthood at 20°C, **(C)** from embryogenesis to Day 1 adulthood at 30°C, and ( **D)** from Day 1 adulthood onward at 20°C. Survival curves compare *Cbr-hsf-1* (RNAi) to control and were analyzed using the Kaplan-Meier log-rank (Mantel-Cox) test. **(E)** Summary table of lifespan metrics corresponding to the data presented in panels B-D. SEM, standard error of the mean; C.I., confidence interval; n, number of animals examined. **(F)** Transcript levels of *CBG19186* in Day 1 adult control (L4440) and *Cbr-hsf-1* (RNAi) worms . Data was analyzed using Student's *t-* test. Data is presented as mean ± SEM. **(G,H)** Normalized transcript counts of *hsf-1* across specific adult stages in **(G)** *C. briggsae* and **(H)** *C. elegans* , based on previously published datasets (see Methods). Independent biological replicates for each time point are shown. The trend line indicates mean values. The Y-axis is displayed on a log scale. **(I)** Survival of Day 1 adult control (L4440) and *Cbr-hsf-1* (RNAi) worms following heat stress exposure. Statistical significance was assessed using the Mann-Whitney test. Bars represent mean ± SD (standard deviation). Total n = 140-154 animals per RNAi condition. **(J)** Survival of Day 1 adult control (L4440) and *Cbr-hsf-1* (RNAi) worms following 24-hour ER stress exposure. Statistical significance was assessed using the Wilcoxon matched-pairs signed-rank test. Bars represent mean ± SD. Total n = 304-347 animals per RNAi condition. Dots on bar graphs represent individual biological batches. For lifespan assays (B-D), 6-8 biological batches were performed per condition. For stress survival assays (I, J), 3-6 biological batches were performed per condition. Asterisks indicate statistically significant differences (** *p* < 0.01, **** *p* < 0.0001).

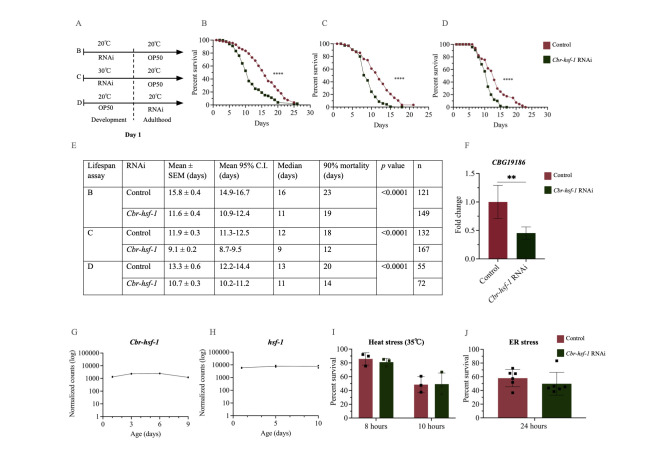



## Description

The nematode *C. briggsae* is commonly used in comparative biological studies alongside its close relative *C. elegans* . We and others have shown that *C. briggsae* exhibits increased thermotolerance relative to *C. elegans* , suggesting that these two species may differ in the mechanisms that regulate stress resistance at elevated temperatures (Felix and Duveau 2012; Jhaveri, et al. 2025; Prasad, et al. 2011).

In *C. elegans* , the heat shock response is regulated by the transcription factor HSF-1 , whose function has been extensively characterized (Morton and Lamitina 2013). Two well described *hsf-1* mutant alleles ( *ok600* and *sy441* ) highlight the essential role of the gene in development and stress regulation. Strong loss of *hsf-1* function ( *ok600* ) causes severe developmental defects that include arrest at the L2-L3 larval stages (Morton and Lamitina 2013). The partial loss-of-function allele ( *sy441* ) and RNAi-mediated knockdown have revealed additional roles in lifespan regulation and stress physiology (Garigan, et al. 2002; Hajdu-Cronin, et al. 2004; Hsu, et al. 2003; Morley and Morimoto 2004; Morton and Lamitina 2013; Volovik, et al. 2012).

Studies on HSF-1 's role in *C. elegans* thermotolerance have reported variable results, which may be in part due to context dependent contribution of the gene to stress resistance. Depending on the age of the animals, the extent of HSF-1 depletion, and assay conditions used, reduced HSF-1 activity has been reported to lower thermotolerance (Finger, et al. 2021; Prahlad, et al. 2008; Steinkraus, et al. 2008), enhance thermotolerance (Golden, et al. 2020), have no effect (Kourtis, et al. 2012; McColl, et al. 2010), reduce thermotolerance after heat pre-treatment (Kourtis, et al. 2012; McColl, et al. 2010), or even enhance survival in young adults immediately after heat shock, with thermotolerance declining with age (Kovacs, et al. 2024). Recent studies have also linked HSF-1 to endoplasmic reticulum (ER) stress responses and survival on tunicamycin, indicating its function extends beyond the canonical heat shock response (Ahmed, et al. 2026; Alcala, et al. 2026; Kovacs, et al. 2024). Together, these findings raise the question of whether the *C. briggsae* *hsf-1* ortholog, *Cbr- hsf-1* plays similar roles in *C. briggsae* .

We recently found that RNAi knockdown of *Cbr- hsf-1* from embryogenesis to Day 1 adult stage caused no obvious phenotype at 20°C but resulted in sterility at 30°C (Jhaveri, et al. 2025). To further investigate the role of *Cbr- hsf-1* , we performed lifespan assays following RNAi-mediated knockdown initiated either during development or adulthood ( Figure 1A ). The developmental knockdown at 20°C caused a significant reduction in lifespan of animals ( Figure 1A, B, E). To determine whether elevated temperature would further compromise survival, the experiment was repeated at 30°C. While the reduction in mean lifespan was similar (27% lower at 20°C and 24% lower at 30°C, compared to controls), population decline was faster at 30°C, as shown by a greater reduction in time to reach 90% mortality (33% at 30°C vs. 17% at 20°C, relative to controls) ( Figure 1E ).

To assess the post-developmental requirements of *Cbr- hsf-1* , RNAi was initiated at 20°C on Day 1 of adulthood. Adult-specific knockdown also significantly shortened the lifespan of animals ( Figure 1D, E), indicating that *Cbr- hsf-1* is required during adulthood for normal lifespan maintenance. Consistent with this, *hsf-1* is expressed in both *C. briggsae* and *C. elegans* adults ( Figure 1G, H). Overall, these results suggest that *Cbr- hsf-1* is required during both development and adulthood for normal lifespan of *C. briggsae* animals.

To verify the effectiveness of *Cbr- hsf-1* RNAi knockdown at 20°C, we measured transcript levels of *CBG19186* , the closest *C. briggsae* ortholog of a small heat shock protein *hsp-16 .2* that is a known target of HSF-1 in *C. elegans* (Jhaveri, et al. 2025; Hajdu-Cronin, et al. 2004; Jones, et al. 1986). Expression of *CBG19186* was significantly reduced in *Cbr- hsf-1* (RNAi) animals relative to controls ( Figure 1F ), consistent with reduced HSF-1 activity. RNAi efficacy at 30°C was supported by the sterility phenotype previously reported for *Cbr- hsf-1* (RNAi) animals (Jhaveri, et al. 2025).

We next assessed *Cbr- hsf-1 's* contribution to acute stress resistance. Following heat shock at 35°C for 8 or 10 hours, or after 24 hours of exposure to tunicamycin, *Cbr- hsf-1* (RNAi) animals exhibited survival comparable to controls ( Figure 1I, J), suggesting that *Cbr- hsf-1* is not required for survival under these assay conditions.

Together, these data show that *Cbr- hsf-1* plays an important role in lifespan maintenance in *C. briggsae* , while having little or no detectable contribution to survival in the acute stress assays tested here. Similar differences between lifespan regulation and acute stress resistance have been reported in *C. elegans* , where reduced HSF-1 activity consistently shortens lifespan but has variable effects on thermotolerance depending on experimental conditions, developmental stage, and degree of knockdown (Morley and Morimoto 2004; Volovik, et al. 2012; Hsu, et al. 2003; Finger et al. 2021; Golden et al. 2020; Kourtis et al. 2012; McColl et al. 2010; Kovacs et al. 2024).

Our results in *C. briggsae* suggest that HSF-1 's role in lifespan maintenance in both species may be at least partially separable from its role in acute stress survival. Because RNAi may not fully deplete *Cbr- hsf-1* activity, it remains possible that stronger loss-of-function approaches could reveal a role in acute stress survival. Further studies using genetic null or strong hypomorphic alleles of *Cbr- hsf-1* will be needed to more comprehensively study its role in *C. briggsae* .

## Methods

**Worm maintenance and RNAi**

Worms were cultured on nematode growth medium (NGM) agar plates seeded with *Escherichia coli* OP50 using standard methods. An RNAi-sensitive *C. briggsae* strain ( JU1018 *mfIs42 [Cel- sid-2 (+); Cel-myo-2 ::DsRed]* ) expressing the *C. elegans sid-2* transgene was used for all experiments (Seetharaman, et al. 2010). RNAi was performed by feeding worms *E. coli* HT115 bacteria that carried double-stranded RNA targeting *Cbr- hsf-1* (Jhaveri, et al. 2025). HT115 bacteria harboring the empty vector (L4440) served as RNAi controls.

**Lifespan**

Embryos were obtained by allowing gravid JU1018 adults to lay eggs for 2-3 hours on the appropriate plates. For developmental knockdown, worms were exposed to RNAi from the embryonic stage and maintained at either 20°C or 30°C until Day 1 of adulthood, after which they were transferred to OP50 seeded NGM plates and maintained at 20°C. For adult-specific knockdown, worms were grown on OP50 plates until Day 1 of adulthood and then transferred to RNAi plates, remaining at 20°C for the duration of the experiment. Animals were scored for survival every 1-2 days by gentle prodding with a platinum wire. Animals that failed to respond were scored as dead. Worms were transferred to fresh plates as needed.

**RT-qPCR**

Approximately 6-8 JU1018 gravid hermaphrodites were placed on RNAi plates seeded with the appropriate bacteria and allowed to lay eggs for 2-3 hours. The adults were then removed, and the progeny were allowed to develop to the Day 1 adult stage. Worms were collected by washing them off plates with M9 buffer and immediately frozen in RNAzol RT (Molecular Research Center, catalog number: RNN190) for RNA extraction. Following RNA extraction, cDNA was synthesized using the LunaScript RT SuperMix Kit (New England Biolabs, catalog number: M3010L), and gene expression was quantified by RT-qPCR using the Bio-Rad cycler CFX 96 and the SensiFAST SYBR No-ROX Kit (FroggaBio, catalog number: BIO-98005). Three independent biological replicates were performed, and results from all replicates were combined for statistical analysis.

*Cbr-iscu-1* was used as the housekeeping gene for normalization. Primer sequences for each gene are listed below.

GL1406 ( *Cbr-iscu-1* ) FP: GCTTCAAATCAGTCTCGCTGC

GL1407 ( *Cbr-iscu-1* ) RP: GTGCCGACGTTCTTGTCGTTT

GL1701 ( *CBG19186* ) FP: CCGTCCAAGACCATTCTCTGT

GL1702 ( *CBG19186* ) RP: ACTGGGAGACGTTGAGGTTG

**Stress assays**

Synchronized populations were generated by allowing gravid JU1018 adults to lay eggs for 2 hours on OP50 seeded NGM plates. Adults were removed after egg laying, and progeny were maintained at 20°C until Day 1 of adulthood.

For heat shock, we followed the previously published protocol from our group (Jhaveri, et al. 2025). Briefly, Day 1 adult control (L4440) and *Cbr- hsf-1* (RNAi) worms were transferred to OP50 seeded NGM plates equilibrated at room temperature (20°C). Plates were then transferred to a 35°C incubator for 8 or 10 hours. After heat treatment, plates were returned to a 20°C incubator, and worms were allowed to recover for 24 hours before survival was scored.

For ER stress, Day 1 adult control (L4440) and *Cbr- hsf-1* (RNAi) worms were exposed to 50 ng/µL tunicamycin (Sigma-Aldrich, catalog number: T7765) in 24-well plates for 24 hours. Following exposure, worms were scored for survival. Animals unresponsive to touch and exhibiting a rigid, rod-like morphology were scored as dead.

***hsf-1* expression**

Gene expression data for *C. briggsae* and *C. elegans* were obtained from published datasets and processed using RNA STAR for alignment and featureCounts for quantification (Schmeisser, et al. 2013; van den Berg and Gupta 2025). Gene counts were normalized in R using DESeq2, and normalized *hsf-1* expression values were extracted for adult time points ( *C. briggsae* : days 1, 3, 6, 9; *C. elegans* : days 1, 5, 10 of adulthood).

**Statistical analysis**

Lifespan data were analyzed using OASIS 2 (Han, et al. 2016). RT-qPCR data was analyzed using Bio-Rad CFX Maestro 3.1 software. All other analyses were performed using GraphPad Prism version 10.6.1. All figures were generated using GraphPad Prism. Statistical tests for each experiment are indicated in the figure legend.
